# Late morphological changes after radiosurgery of brain arteriovenous malformations: an MRI study

**DOI:** 10.1007/s00701-016-2876-3

**Published:** 2016-07-01

**Authors:** Hana Malikova, Eva Koubska, Zdenek Vojtech, Jiri Weichet, Martin Syrucek, Jan Sroubek, Aaron Rulseh, Roman Liscak

**Affiliations:** 1 Department of Radiology, Na Homolce Hospital, Roentgenova 2, 150 00 Prague, Czech Republic; 2 Institute of Anatomy, Second Medical Faculty, Charles University in Prague, U Nemocnice 3, 120 00 Prague, Czech Republic; 3 Department of Neurology, Na Homolce Hospital, Roentgenova 2, 15000 Prague, Czech Republic; 4 Department of Neurology, 3rd Medical Faculty, Charles University in Prague, Ruska 87, 10000 Prague, Czech Republic; 5 Department of Pathology, Na Homolce Hospital, Roentgenova 2, 15000 Prague, Czech Republic; 6 Department of Neurosurgery, Na Homolce Hospital, Roentgenova 2, 15000 Prague, Czech Republic; 7 Department of Stereotactic and Radiation Neurosurgery, Na Homolce Hospital, Roentgenova 2, 15000 Prague, Czech Republic

**Keywords:** Gadolinium enhancement, Wallerian degeneration, Cyst formation, Post-irradiation

## Abstract

**Background:**

Radiosurgery by Gamma Knife (GK) is an effective treatment for brain arteriovenous malformations (AVM). The aim of the present study was to evaluate late, radiation-induced changes detectable by MRI after AVM radiosurgery in patients treated minimally 10 years prior, with AVM obliteration proven by angiography.

**Methods:**

Thirty-five patients with 37 AVMs were included. AVMs were irradiated 16.6 ± 3.5 years prior with AVM obliteration proven 13 ± 4 years prior. All patients underwent recent MRI examinations, including application of gadolinium-based contrast.

**Results:**

In one case, post-irradiative cystic formation with mass effect and signs of hemorrhage requiring surgery was found. Post-gadolinium enhancement at the site of obliterated nidi was apparent in 28 of 37 cases (76 %). In all cases except one, the mean volume of enhancement at the time of review was clearly lower than the volume of the originally irradiated AVM (88 ± 20 %; median 92 %); in one case the extent was 142 % greater than the irradiated AVM. When we compared enhancing and non-enhancing nidi, we found that enhancing nidi were significantly larger than non-enhancing nidi at the time of radiosurgery (4.39 ± 3.35 cc vs. 0.89 ± 0.79 cc,* p* = 0.004). Enhancement was not influenced by total radiation dose, patient age at the time of irradiation, duration since radiosurgery, or the number of irradiations. Wallerian degeneration was found in nine of 37 cases (24 %); in six cases the optical tracts were affected and visual field defects were proven. In five of nine cases (55.6 %) with Wallerian degeneration previous hemorrhage was present. Dual vascular pathology was found in eight of 35 patients (23 %).

**Conclusions:**

GK radiosurgery for AVM is a safe treatment method although delayed complications may occur. Post-gadolinium enhancement of obliterated nidi may indicate an active post-irradiative process.

## Introduction

Arteriovenous malformations (AVM) in the brain are characterized by a conglomerate of tortuous vessels with feeding arteries and draining veins. These vessels shunt directly from the arterial to the venous system; normal intervening capillary networks are absent. Additionally, the endothelial cell layer in AVMs structurally differs from normal cerebrovascular endothelium [1]. Hemorrhage associated with AVM is the most significant cause of morbidity and mortality. Treatment for AVM includes surgery, radiosurgery, or embolization alone or in combination. Radiosurgery by Gamma Knife (GK) has been proven an effective treatment for AVM [2–6].

We have provided stereotactic radiosurgery for the treatment of AVM at our institution since 1992. We use digital subtraction angiography (DSA) and magnetic resonance imaging (MRI) for the stereotactic localization of AVMs. First MRI follow–up is standardly performed 2 years after radiosurgery. After the disappearance of flow voids on MRI, DSA is indicated to prove complete AVM obliteration. In the case of suspected complication, computed tomography (CT) or MRI is performed to exclude re-bleeding or postirradiation edema. Once complete obliteration of the AVM is verified by DSA, only symptomatic patients continue to be followed actively at our institution.

It has previously been reported that AVM obliteration was achieved in 74 % patients after the first round of radiosurgery and in 69 % of remaining patients after a second round of radiosurgery [6]. The overall chance of cure was 92 % [6]. The risk of re-bleeding after radiosurgery has been reported as 2.1 % annually until full obliteration and the overall mortality from re-bleeding as 1 %, with cumulative morbidity risk at 3.4 % [6].

Although stereotactic radiosurgical treatment for AVM is a well-established method, delayed, long-term postirradiative changes on MRI after GK treatment have not been sufficiently investigated. In the present study, we evaluated late radiation-induced changes on MRI following radiosurgical AVM treatment in a group of patients treated between the years of 1993 and 2005 in whom complete AVM obliteration was proven by DSA, with no symptomatic complications since.

## Materials and methods

### Patient selection

We examined all available medical records of patients treated by GK radiosurgery for AVM between the years 1993 and 2005. In this period, 675 patients were identified. They came from the region of former Czechoslovakia and also abroad. Only patients living in the vicinity of our facility were selected for accessibility purposes. We further excluded all patients who underwent open surgery following GK radiosurgery failure, as well as patients with severe disabilities, pregnant or lactating women, and patients older than 70 years. One hundred and eight patients were contacted by phone or by mail and offered a follow-up MRI examination with gadolinium-based contrast. The study was approved by the local ethics committee and all participants provided written informed consent.

Summary of the inclusion criteria:

1. Patients that underwent their first radiosurgical procedure for AVM between the years 1993 and 2005 (minimally 10 years prior to the present study) by Leksell Gamma Knife (Elekta Instruments AB, Stockholm, Sweden). Patients that underwent a second or third radiosurgical procedure 3 years after the first procedure due to patent AVM were also included.
2. Complete obliteration of the AVM proven by DSA.
3. Age under 70 years, not pregnant, not lactating, and in good health.
4. No contraindications to MRI.

### MRI methods

All patients underwent MRI examinations. MRI was performed using a 3T whole-body MRI scanner (Magnetom Skyra, Siemens, Erlangen, Germany) fitted with a 32-channel head coil. The MRI protocol included the following sequences and parameters:

- TSE T2/PD transversal scans (voxel size 0.3 × 0.3 × 0.4 mm, slices 27, distance factor 30 %, slice thickness 4 mm, TR 3600 ms, TE 9.4 ms, averages 1, concatenations 1, flip angle 160°)
- FLAIR FAT-SAT transversal scans (voxel size 0.7 × 0.7 × 3.5 mm, slices 36, distance factor 20 %, slice thickness 3.5 mm, TR 8500 ms, TE 81 ms, averages 1, concatenations 2, TI 2439 ms, flip angle 150°)
- T2 SWI 3D transversal scans (voxel size 0.5 × 0.5 × 2.5 mm, slabs 1, slices per slab 64, distance factor 20 %, slice thickness 2.5 mm, TR 27 ms, TE 20 ms, averages 1, concatenations 1, flip angle 15°)
- GE T1 coronal and transversal scans (voxel size 1.0 × 1.0 × 3.0 mm, slices 22, distance factor 0 %, slice thickness 3 mm, TR 3 ms, TE 168 ms, averages 3, concatenations 2, flip angle 50°)
- SE T1 sagittal scans (voxel size 0.4 × 0.4 × 4.0 mm, slices 25, distance factor 30 %, slice thickness 4 mm, TR 650 ms, TE 8.2 ms, averages 2, concatenations 1, flip angle 50°)
- DWI FAT-SAT transversal scans (voxel size 0.6 × 0.6 × 4 mm, slices 25, distance factor 30 %, slice thickness 4 mm, TR 6400 ms, TE 98 ms, averages 3, concatenations 1, diffusion weightings 2, b = 0, b = 1000, ADC maps)
- GE T1 3D after gadolinium intravenous administration (voxel size 0.7 × 0.7 × 0.7 mm, slabs 1, slices per slab 240, distance factor 50 %, slice thickness 0.7 mm, TR 1820 ms, TE 2.37 ms, averages 2, concatenations 1, TI 1070 ms, flip angle 6°).

Volumetry of the enhancing AVM nidus was performed, when present. Volumes were segmented manually and calculated with software developed on in-house for a standard work console.

The defined endpoints of interest were as follows:

A. In–field changes (in the field of radiosurgery)
  Cyst formation was considered relevant in cases where minimally one of the following was present: mass effect, high protein content, perifocal vasogenic edema, cyst wall enhancement. Post-hemorrhagic pseudocystic changes with cerebrospinal fluid content, without enhancement and without mass effect were considered as insignificant post-hemorrhagic changes.
  Presence of post-gadolinium AVM nidus enhancement including volumetry.
B. Out-of-field changes
  Presence of Wallerian degeneration due to irradiation or complication of hemorrhagic AVM.
C. Dual pathology elsewhere
  Presence of vascular or other dual pathology on MRI.

### Statistics

Data were expressed as mean ± SD in variables with Gaussian distribution and as median. We analyzed various potential effects on post-gadolinium nidus enhancement by means of unpaired* t* tests; categorical values were analyzed using the Chi-square test;* p* values ˂0.05 were considered significant. Analyses were performed using STATISTICA software, vers. 12.

## Results

### Patient selection

The patient response rate was 32.4 %. Thirty-five patients (14 male, 21 female) met the inclusion criteria and were included in the study; mean age of included subjects was 49.4 ± 12.4 years (median, 46 years). The AVMs were irradiated 16.6 ± 3.5 years (median, 17 years, range, 10–22 years) prior; AVM obliteration proven by DSA performed 13 ± 4 years (median, 13 years, range, 4–21 years) prior. Thirty-seven AVM nidi in 35 subjects were irradiated (two patients with two AVMs). AVMs were graded according to the Spetzler–Martin AVM classification [7] as Grade I in five, Grade II in 21, Grade III in eight, and Grade IV in three cases. The mean volume of the irradiated nidi was 3.41 ± 3.27 cc (median, 2.60 cc). Eleven AVM nidi were irradiated twice and 1 AVM was irradiated three times. The maximum dose of the first irradiation was 43.5 ± 7 Gy (median, 44 Gy, range, 34–54 Gy) with a margin dose of 21.7 ± 3.6 Gy (median, 22 Gy, range, 17–27 Gy). Eleven AVMs were secondarily irradiated with a maximum dose of 38.5 ± 7 Gy (median, 40 Gy, range, 28–46 Gy) and a margin dose of 19.2 ± 3.5 Gy (median, 20 Gy, range, 15–25 Gy). One AVM was irradiated a third time by a maximum dose of 44 Gy and a margin dose of 22 Gy.

### MRI data

Only one (of 37 irradiated nidi; 2.7 %) serious late complication requiring urgent surgery was found on MRI; it was a post-irradiation cyst formation with mass effect and signs of hemorrhage. For more details, see the case report below.

We found next 12 pseudocysts with hemosiderin rim surrounded by gliosis, with cerebrospinal fluid content. Eleven of those pseudocysts were present on MRI before GK treatment; all those AVMs manifested as intracranial bleeding. In one patient, clinical symptomatic bleeding was present 1 year after irradiation, since that time pseudocystic changes were present. Those cases were considered as irrelevant post-hemorrhagic pseudocystic changes.

**Fig. 1:**
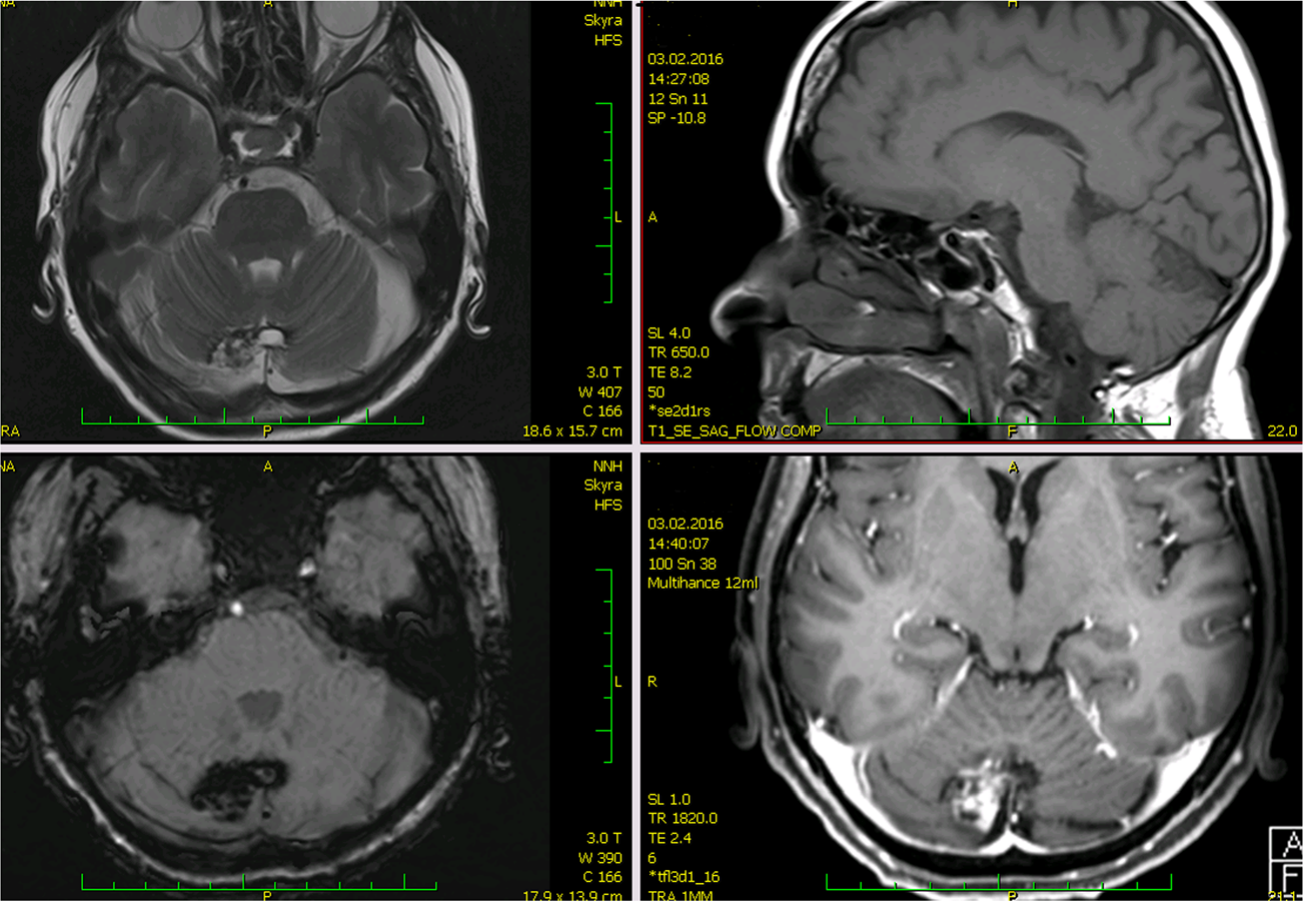
MRI appearance of the cavernous malformation-like lesion. AVM was irradiated 18 years prior; at the time of irradiation, the AVM nidus was 0.71 cc, AVM obliteration was proven 3 years later. There were no signs of hemorrhage. Recent MRI showed extended hemosiderin deposition and post-gadolinium enhancement covering a larger area than the AVM. (*Upper left*: TSE T2 transversal scan,* upper right*: SE T1 sagittal scan,* lower left*: T2 SWI 3D transversal scan,* lower right*: GE T1 3D post-gadolinium transversal scan)

Post-gadolinium enhancement of the obliterated AVM nidus site was apparent in 28 of 37 cases (76 %). In only nine of 37 (24 %) cases, enhancement of the AVM nidus site was not detectable after intravenous contrast administration. In all cases except one, the mean volume of enhancement was clearly lower than the volume of the irradiated AVM. The extent of enhancement was approximately 88 ± 20 % (median 92 %) less than the volume of originally irradiated nidus. In one case, the extent of enhancement was clearly greater than the irradiated nidus (roughly 142 %) and was surrounded by irregular deposits of hemosiderin, although the patient did not suffer from hemorrhage before radiosurgery or at time of the last MRI follow-up 15 years prior. The MRI appearance in this case resembled cavernous malformation (Fig. 1).

Enhancing nidi were significantly larger than non-enhancing nidi at the time of irradiation (4.39 ± 3.35 vs. 0.89 ± 0.79 cc,* p* = 0.004). For more details, see Table 1. Post-gadolinium enhancement was not influenced by any of the following: total radiation dose, patient age at the time of irradiation, time delay since GK radiosurgery. When we compared subgroups, AVM nidi irradiated once versus nidi irradiated more than once (two or three times), we did not find any statistically significant differences in proportion of enhancement: 17 of 25 (68 %) AVM nidi irradiated once and 10 of 11 (91 %) nidi irradiated more than once enhanced after gadolinium administration (*p* = 0.14, Chi-square test).

**Table 1:** Post-gadolinium enhancement of AVM nidi

Volumes of irradiated nidi (cc)	Presence of post-gadolinium enhancement	Presence of clinically significant or MRI detectable hemorrhage before AVM occlusion
>10.00	100 %	0 %
5.00–10.00	100 %	17 %
1.00–5.00	85.7 %	64 %
<1.00	30 %	50 %

*AVM* arteriovenous malformation,* MRI* magnetic resonance imaging

Table 1 also documents that AVMs with nidi larger than 5.00 cc were only occasionally associated with hemorrhage. These nidi manifested with intracranial bleeding in 12.5 % of cases; significantly less (*p* = 0.04) than nidi smaller than 5.00 cc, in which hemorrhage prior to radiosurgery was detected in 54.2 % (see Table 1 for comparison of AVM-associated hemorrhage before radiosurgery in relation to AVM nidus volume).

**Table 2:** Summary of MRI findings

		AVM without pre-treatment hemorrhage	AVM with pre-treatment hemorrhage
No. of nidi		19	18
Clinically relevant cyst formation		0	1
Wallerian degeneration	Optical tract	2	4
	Pyramidal tract	1	
	Corpus callosum		1
	External capsule	1	
Cavernous malformation-like MRI appearance		1	0
Delayed nidus enhancement		14	14
Vascular dual pathology		Cavernous malformation in 1 pt; Multiple venous angiomas in 1 pt	Multiple cavernous malformations in 1 pt; Arterial aneurysms in 4 pts; Venous angioma in 1 pt
Non-vascular dual pathology		LGG in 1 pt	Multiple metastases in 1 pt

*MRI* magnetic resonance imaging,* LGG* low-grade glioma

**Fig. 2:**
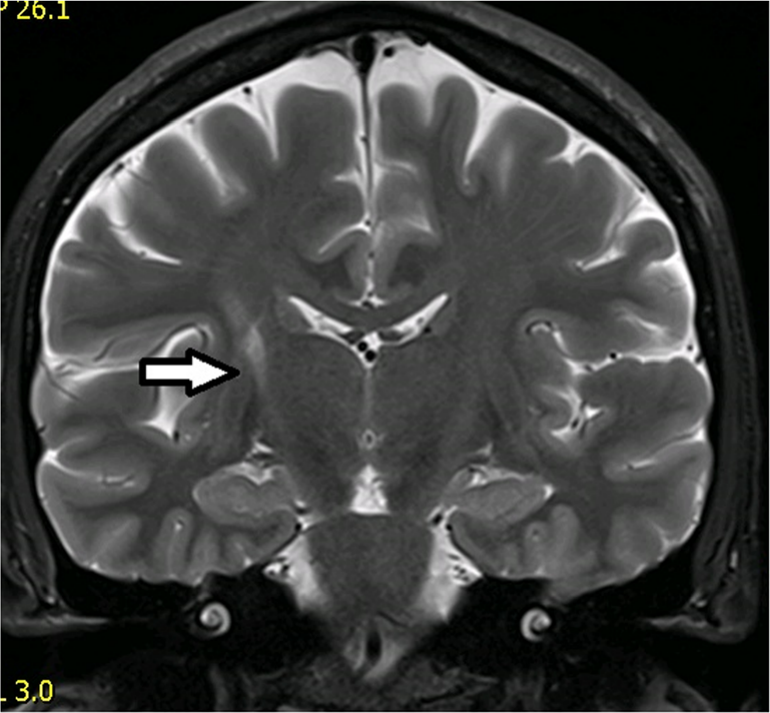
Wallerian degeneration of the right pyramidal tract. The irradiated AVM was situated in the right centrum semiovale. The degenerated pyramidal tract is depicted by the* white arrow* (*TSE T2* coronal scan)

Wallerian degeneration was found in nine of 37 cases (24 %). In six cases, the optical tracts were affected (in four cases AVMs were situated in the temporal lobes, in two cases in temporo-occipital regions). In one case, the pyramidal tract was degenerated (AVM was situated in the centrum semiovale; Fig. 2) and in one case Wallerian degeneration affected the external capsule and corpus callosum. In all patients with signs of Wallerian degeneration of the optical tracts, visual field defects were proven. In five of nine cases (55.6 %), previous hemorrhage before treatment was present. For more details, see Table 2.

No dual pathology was located in the field of radiosurgery. With the exception of two patients who suffered dual AVM, vascular dual pathology was present in eight of 35 patients (23 %); in four subjects arterial aneurysms, in two subjects cavernous malformations, and in two patients venous angiomas were detected. Non-vascular dual pathology was detected in two patients (low-grade glioma in one patient and multiple metastases from breast carcinoma in one patient). Multiple metastases were found at time of the late review and the patient was immediately sent for oncological treatment. In the second case, the presence of low-grade glioma was known since the time of AVM diagnosis. The patient was sent for MRI after the first generalized epileptic seizure; AVM in the left occipital lobe and low-grade glioma in the right frontal lobe was found. In the same year, the patient underwent neurosurgical resection of the glioma and radiosurgery for the AVM. At time of the late review, the patient was asymptomatic (for more details, see Table 2).

### Case report

**Fig. 3:**
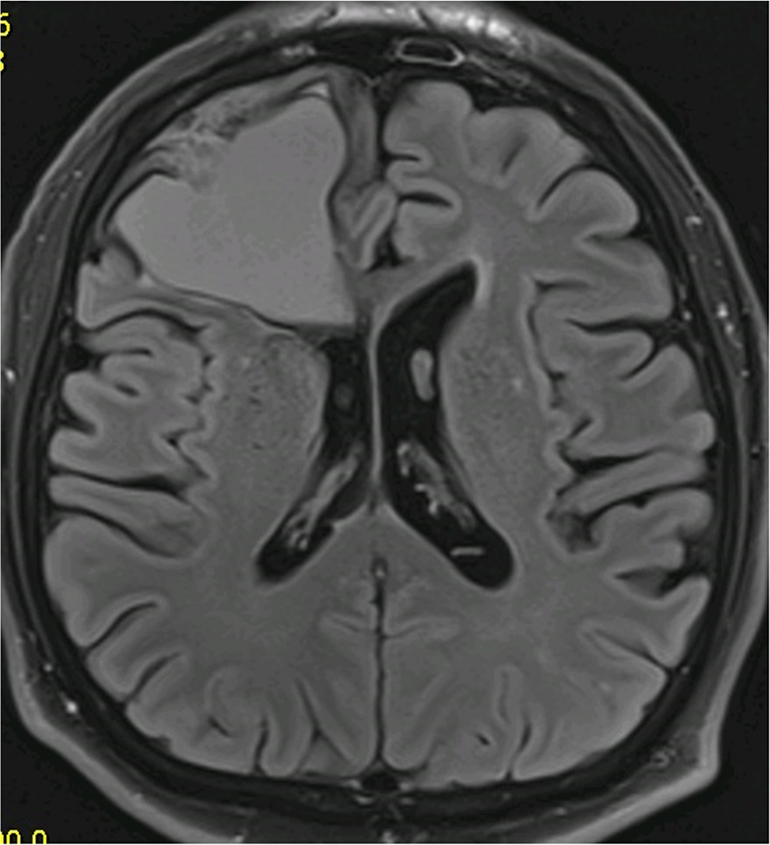
Cyst formation in the right frontal lobe (non-contrast scan). There are clear signs of mass effect, high protein cyst content, and fluid–fluid level (FLAIR transversal scan)

**Fig. 4:**
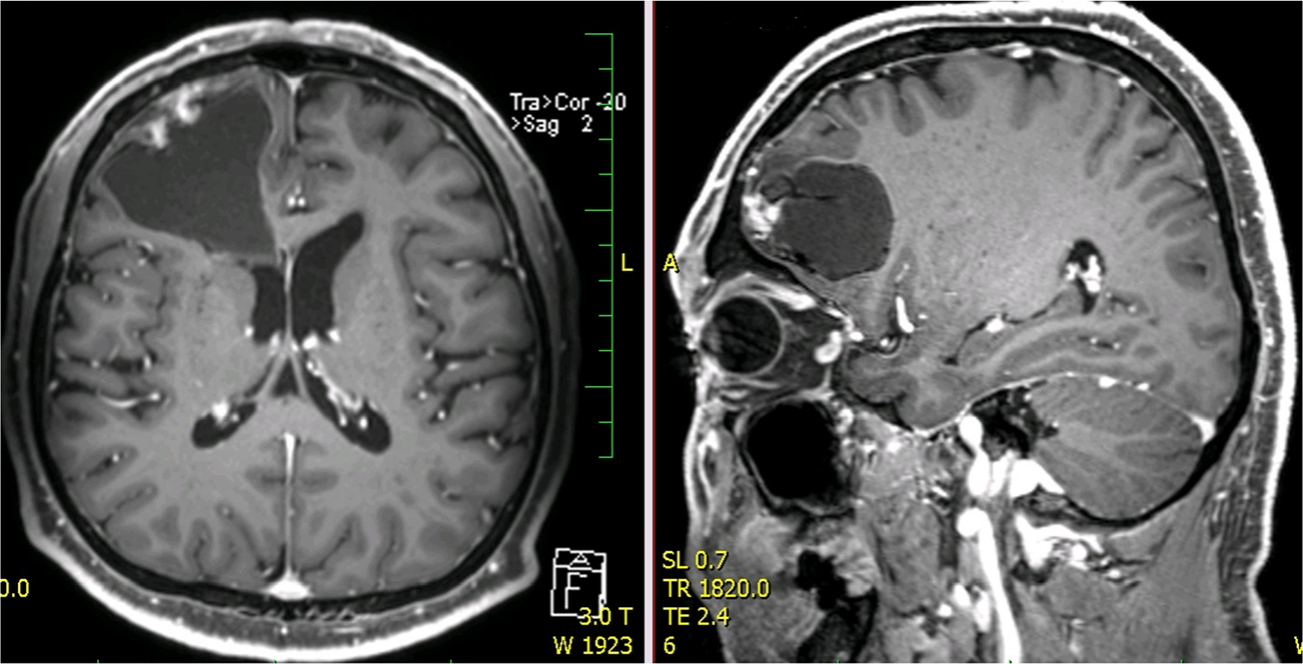
Cyst formation in the right frontal lobe (post-gadolinium scan). Nodular irregular enhancement in the wall of the cystic formation is present (GE T1 3D post-gadolinium transversal and sagittal scans)

**Fig. 5:**
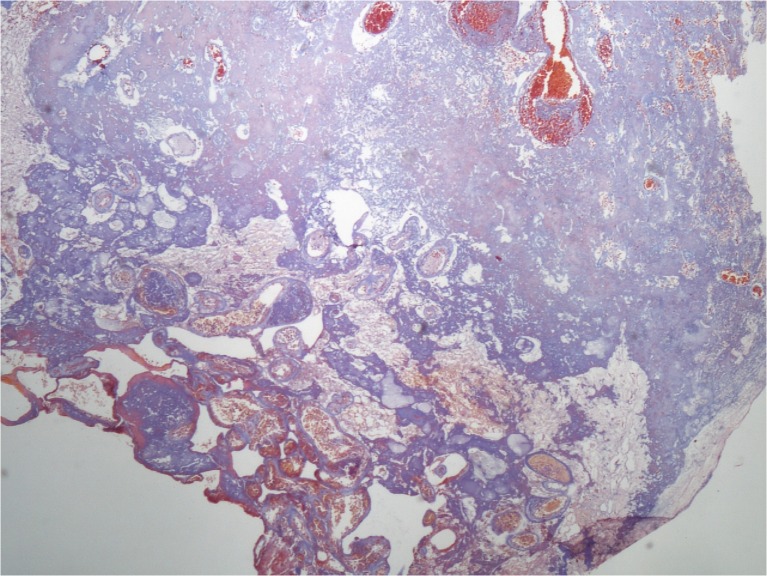
Histology sample 1. AVM with occluded channels and channels with signs of recanalization and neoangiogenesis; surrounding gliosis is also remarkable (Masson trichrome – elastica, magnification 40×)

**Fig. 6:**
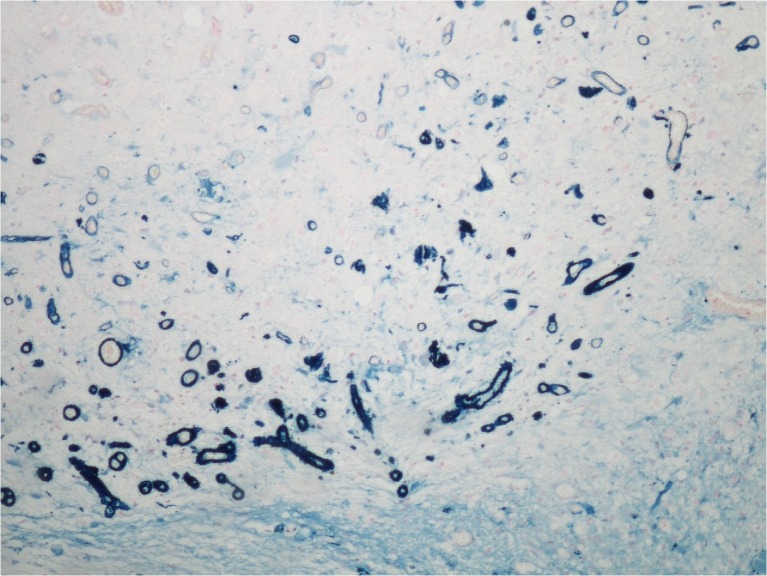
Histology sample 2. Deposition of ferric compounds were detected in blood vessel walls (Prussian Blue, magnification 100×)

Twenty-one years ago, a 60-year-old man underwent radiosurgery for an AVM in the right frontal lobe, which manifested with intracerebral hemorrhage. The size of the nidus was 12 × 24 × 13 mm (nidus volume was 1.95 cc), and the AVM was irradiated by a maximum dose of 50 Gy with a marginal dose of 25 Gy. One year later, DSA follow-up showed complete obliteration of the AVM. The patient did not suffer from any clinical manifestations and therefore follow-up at our institution was terminated; he was advised to continue follow-up with his local neurologist. Twenty years later, he agreed to participate in the present study. MRI revealed cystic changes measuring 44 × 51 × 39 mm in the right frontal region, with defiguration of the frontal horn of the right lateral ventricle and slight midline shift. Signs of repeated hemorrhage with fluid–fluid level were observed (Fig. 3). After intravenous contrast administration, the wall of the cyst and the previously irradiated AVM nidus clearly enhanced (Fig. 4). Neurologic examination revealed only prefrontal psychological changes. The patient was informed that surgery was needed and provided consent. Two weeks later, he was admitted to our hospital following a secondary generalized epileptic seizure (negative history) with subsequent prolonged unconsciousness, for which he was intubated. CT showed cyst formation detected previously on MRI, DSA proved AVM occlusion. After extubation, four electrographic seizures originating in right prefrontal leads were found on routine 20-min EEG. Interictally, bifrontal nonspecific abnormalities with left preponderance were present. Antiepileptic drugs (phenytoin, clonazepam) were introduced. On subsequent neurologic examination, prefrontal psychological changes were again found. Urgent surgery was indicated. A small frontal craniotomy via right supraorbital incision was performed. Linear incision of the dura was followed by microsurgical resection of the MRI enhancing lesion located superficially. The lesion had a fibrotic consistency and several small vessels were seen on its surface. These vessels tightly adherent to the cyst were coagulated and cut. During the resection of the enhancing lesion, the cyst was entered and a yellowish liquid was drained. Communication with the ventricle appeared spontaneously and the ostium was dilated deliberately. Surgery was proceeded by hemostasis and dural watertight suturing. The bone flap was fixed by a titanium plate and the soft tissue was sutured in layers. Histological examination of the resected sample revealed AVM with occluded channels, channels with recanalization, and signs of neoangiogenesis (Fig. 5). Surrounding gliosis was remarkable. In the vessel walls, deposition of ferric compounds was detected (Fig. 6).

## Discussion

Radiosurgery is a very successful minimally invasive treatment for brain AVM, with AVM obliteration occurring in more than in 90 % of treated cases [6]. The disadvantage of this method is the risk of re-bleeding until complete obliteration, as well as possible acute and subacute complications of GK treatment such as postirradiative edema [6]. However, there is also risk of delayed complications due to radiosurgical injury [8]. Radiation injury depends on numerous factors, of which the dose and the volume of irradiated tissue are the most important [9]. However, it is well known that the degree of radiographic changes is extremely variable and unpredictable [10]. It is influenced by the type of tissue treated, by the location of the lesion, as well as by individual sensitivity to radiation injury. It is widely accepted that normal brain tissue is more vulnerable to radiation injury than brain tumors [11].

In the present study, 35 patients (with 37 AVMs) were included who were treated by radiosurgery 16.6 ± 3.5 years prior to study onset, with AVM obliteration proven 13 ± 4 years prior. We found only one case (2.7 %) of serious late post-irradiative complication; a large cystic mass with high protein content and an enhancing wall. This patient required open surgery. Cyst formation is a rare but well-documented complication following radiosurgery for AVM [12, 13]. Its prevalence varies according to published reports from 1.1 % [14] to 5.5 % [13]. The prevalence of cyst formation may be higher in unruptured AVMs, as normal perinidal brain parenchyma is more vulnerable to radiation than gliosis after hemorrhage [14]. However, in the present case, symptomatic bleeding was present before GK radiosurgery. In a previous study by Izawa et al., predisposing factors for cyst formation included a large nidus volume, a high maximum dose, lobar localization, and complete AVM obliteration [15]. Delay from radiosurgery to cystic formation ranged from several months to many years. In the present case, GK radiosurgery was performed 21 years prior and AVM obliteration was proven 20 years prior to the study.

According to previous reports, there are two types of cystic formations: chronic encapsulated expanding hematoma, and cysts with a neo-angiogenetic nodule in their wall. Common etiological mechanisms have been suggested for both types of lesions [16]. The development of cystic changes may occur in several steps: inflammation of the perinidal parenchyma; blood–brain barrier damage due to inflammation; formation of dilated capillary vessels with damaged walls; serum protein exudation with subsequent development of a perinidal cavity; neovascularization in the wall of this cavity with neo-angiogenetic nodule formation; repeated hemorrhage from fragile vessels into the cavity leading to osmotic cyst expansion or chronic encapsulated hematomas.

In the present study, we found that most obliterated nidi (28 of 37 nidi; 76 %) still clearly enhanced after intravenous gadolinium contrast administration, 16.6 ± 3.5 years after GK radiosurgery. This is in agreement with a similar study conducted by Kihlstrom et al., where 61 % of obliterated nidi enhanced after contrast administration [17]. They examined 18 patients with AVMs, who were treated with GK and in whom MRI was obtained a mean of 14 years after treatment and 10 years after confirmed AVM obliteration [17]. Postgadolinium enhancement in the site of AVM nidus is explainable by histological findings that were gained in a surgically treated patient who was discussed above. Histological evaluations showed signs of AVM vascular channel recanalization. This may explain the enhancement detected in the AVM nidus site, however it does not indicate the presence of an arteriovenous shunt, which was clearly excluded by DSA. Histology also showed newly formed vascular structures with iron deposits in their walls. These findings correspond with neo-angiogenesis and damage to the walls of newly formed vessels. Vascular injury, damage to the blood–brain barrier, vasogenic edema, and neo-angiogenesis are well-known effects of radiosurgery. Neovascularization has been described after radiosurgery for cavernous malformations [18] as well as radiosurgery in hippocampal sclerosis [8].

When we compared enhancing and non-enhancing nidi, we found that later enhancing nidi were significantly larger than non-enhancing at time of radiosurgery (4.39 ± 3.35 cc vs. 0.88 ± 0.79 cc,* p* = 0.004). However, enhancement was not influenced by total radiation dose, patient age, or duration since GK radiosurgery. We also did not find any statistical differences between postgadolinium nidi enhancement in subgroups AVM nidi irradiated once and more than once (two or three times). We found that nidi larger than 5.00 cc were associated with significantly less pre-treatment hemorrhage (12.5 % nidi) as opposed to nidi smaller than 5.00 cc (54.2 %). Similar findings have been described previously [6, 19, 20].

We detected Wallerian degeneration in nine of 37 cases (24 %). Typically, the optical tracts were affected in cases where the irradiated AVM was situated in temporal or occipital lobes. These patients suffered from visual field defects. Wallerian degeneration is a complication of GK radiosurgery that has received little attention previously [21]. However, in the case of AVM, Wallerian degeneration may also by partly or fully caused by previous hemorrhage. Fifty-five percent of subjects with signs of Wallerian degeneration had hemorrhage as a manifestation of AVM.

Our study has several important limitations. First, not all patients treated between the years 1993 and 2002 could be evaluated; the patient response rate was only 32 %. Most patients were lost to follow-up while only some refused to participate in the study. Due to the low response rate, it is difficult to assess sensitivity of the MRI findings. We could speculate that the low response rate may be partially due to good medical condition of the patients or on the contrary due to bad health. Some patients probably died. Also, in some older cases, medical documentation was not complete. We included patients irradiated only once, as well as those who underwent 2–3 irradiations. This approach may have resulted in some bias. However, we did not observe significant differences in the proportion of postgadolinium enhancement between these two subgroups.

In conclusion, GK radiosurgery for AVM is a safe treatment method, although delayed complications cannot be avoided and were found 16.6 ± 3.5 years after therapy. Additionally, post-gadolinium enhancement could be a sign of an active, delayed post-irradiative process.
